# Health-promoting behaviors are associated with lower depression and anxiety in Ecuadorian medical residents: a cross-sectional study

**DOI:** 10.3389/fpubh.2025.1715795

**Published:** 2026-01-12

**Authors:** Xavier Sánchez, Andrés Cruz, María Daniela Cortez, Ruth Jimbo-Sotomayor, Santiago Escalante, Diego Mera

**Affiliations:** 1 Community and Primary Care Research Group – Ecuador (CPCRG-E), Centro de Investigación para la Salud en América Latina (CISeAL), Pontificia Universidad Católica del Ecuador (PUCE), Quito, Ecuador; 2 Pontificia Universidad Católica del Ecuador (PUCE), Quito, Ecuador

**Keywords:** anxiety, depression, Ecuador, health behavior, mental health

## Abstract

**Introduction:**

Mental health problems, hazardous drinking, and unhealthy behaviors are global concerns among medical residents, particularly in resource-limited regions such as Latin America. Long hours, heavy workloads, and stigma increase the risk of depression, anxiety, and substance misuse. This study assessed the prevalence of mental health symptoms, alcohol use, and health behaviors among residents in Ecuador.

**Methods:**

A cross-sectional online survey was conducted among 307 residents at the Pontificia Universidad Católica del Ecuador, after inviting all 1,122 enrolled residents to participate. Validated instruments assessed depression (PHQ-9), anxiety (GAD-7), hazardous drinking (AUDIT), and health behaviors (Health Behavior Inventory, HBI), alongside sociodemographic and training factors. Prevalence rates were estimated, and associations were analyzed using correlations and mixed-effects logistic regression.

**Results:**

The prevalence of depression, anxiety, and hazardous drinking was 38.8, 33.9, and 12.1%, respectively. Depression and anxiety were strongly correlated and inversely associated with health-promoting behaviors. Higher HBI scores significantly reduced the odds of depression (OR = 0.34, 95% CI 0.24–0.47, *p* < 0.001) and anxiety (OR = 0.52, 95% CI 0.39–0.69, *p* < 0.001). Hazardous drinking was independently associated with surgical programs and tobacco use.

**Conclusion:**

Ecuadorian residents face substantial burdens of depression, anxiety, and hazardous drinking. Health-promoting behaviors were associated with better mental health, underscoring the importance of targeted interventions, workload reforms, and stigma reduction. From a public health perspective, supporting residents’ well-being is critical not only for individual outcomes but also for patient safety and the sustainability of healthcare systems.

## 1 Introduction

Mental health challenges, hazardous drinking, and poor health behaviors among medical trainees are critical global public health concerns, affecting both individual well-being and healthcare quality. The intense demands of medical training—long hours, high-stakes clinical duties, and emotional stress—place residents at a heightened risk for depression, anxiety, and substance misuse (1). Systematic reviews estimate that 27–40% of medical students worldwide experience depression, underscoring the urgency of addressing these issues (2). Beyond personal consequences, untreated mental health conditions can compromise patient care through increased medical errors and diminished empathy (3).

Resident physicians with depressive symptoms are significantly more likely to make medication errors, while burnout has been linked to higher rates of patient safety incidents (3, 4). Hazardous drinking, often a stress-coping mechanism, further exacerbates these risks by impairing decision-making and increasing burnout rates among trainees (5). These issues are particularly concerning in resource-limited settings, where the well-being of residents is essential for maintaining safe and effective patient care.

Despite increasing global awareness, research on medical trainees’ mental health remains disproportionately focused on high-income countries, with limited data from Latin America, particularly Ecuador. The World Health Organization (WHO) reports that low- and middle-income countries allocate only 2% of their health budgets to mental health services, highlighting the region’s structural limitations (6). Additionally, cultural attitudes toward alcohol and pervasive stigma surrounding mental health may heighten risks for trainees (6). Intense clinical rotations, frequent night shifts, and inadequate mental health support in Latin American medical residency training settings may amplify these challenges (7).

In Ecuador, medical specialty training is not nationally standardized; each institution designs programs to meet local needs. Residents rotate mainly in public hospitals, working about 80 h per week—80% clinical duties (including night shifts every fourth day) and 20% academic activities—per national regulations (8). This demanding schedule highlights the pressures residents face, framing the need to examine their mental health and behaviors.

Although mental health problems among medical trainees have been widely studied globally, there is a lack of empirical data on depression, anxiety, hazardous alcohol use, and health-promoting behaviors among Ecuadorian medical residents. This absence of local evidence is critical given Ecuador’s broader mental health challenges, persistent stigma, and limited support (9), which also affect residency training. This study addresses this gap by providing empirical data on the mental health and health-related behaviors of Ecuadorian medical residents. Specifically, it aimed to assess the prevalence and severity of depression, anxiety, and hazardous drinking, as well as health-promoting behaviors, and to explore their associations with training program characteristics and sociodemographic factors. Establishing this baseline offers actionable insights to improve resident well-being and to inform mental health policies in Ecuador.

## 2 Materials and methods

### 2.1 Study design and setting

This cross-sectional study was conducted among residents at the Pontificia Universidad Católica del Ecuador (PUCE), the country’s leading residency training institution. PUCE offers 18 specialty programs distributed across its seven campuses and is recognized for its extensive clinical and surgical training nationwide. Data collection took place from February to March 2025, intentionally scheduled during a standard academic term to capture typical patterns of mental health and health-related behaviors, avoiding periods of heightened academic stress such as examinations.

### 2.2 Sample

The target sample size was based on the 1,122 residents enrolled in 2025. Assuming a 95% confidence level, 5% margin of error, and *p* = 0.5 (maximizing variance given no prior data), the finite population formula (10), $n=\frac{Z^{2}*p*(1-p)*N}{E^{2}*(N-1)+Z^{2}*p*(1-p)}$ , where *Z* = 1.96 (95% CI), *p* = 0.5, *E* = 0.05, and *N* = 1,122, yielded a minimum sample size of approximately 287 residents. Recruitment invitations were sent to all 1,122 eligible residents, and 307 residents consented and completed the survey. This exceeded the minimum requirement, thereby increasing precision and reducing the margin of error from 5% to approximately 4.7%.

### 2.3 Participants

The study included 307 residents across training years 1–4 from 18 programs, categorized as surgical (e.g., Anesthesiology, General Surgery, Orthopedics) or clinical (e.g., Internal Medicine, Pediatrics, Geriatrics) based on operative focus. Inclusion criteria were active enrollment in a PUCE residency program during the study period, ability to complete the online survey, and provision of informed electronic consent. Exclusion criteria included residents who were on academic or medical leave during data collection, those temporarily completing external or out-of-country rotations, and incomplete surveys (none were recorded). Ethical approval was granted by the PUCE Human Research Ethics Committee (EO-071-2024, V2).

### 2.4 Instruments

Data were collected using validated self-report instruments administered through an online survey in Spanish. Depression was measured with the Patient Health Questionnaire-9 (PHQ-9), a 9-item scale (range 0–27) assessing depressive symptoms over the previous 2 weeks; scores ≥10 indicate major depression with 88% sensitivity and specificity (11, 12). In this study, internal consistency was excellent (Cronbach’s α = 0.93). The Spanish version used has demonstrated strong psychometric properties in Ecuadorian populations (13, 14). Anxiety was measured using the Generalized Anxiety Disorder-7 (GAD-7) is a 7-item tool (range 0–21) for assessing anxiety severity, with scores ≥10 denoting moderate-to-severe symptoms, supported by 89% sensitivity and 82% specificity (15). Its Spanish version has been validated in multiple Latin American samples, including Ecuador (16, 17). Internal consistency in this sample was excellent (α = 0.92). Hazardous drinking was assessed using the Alcohol Use Disorders Identification Test (AUDIT), a 10-item instrument (range 0–40) with scores ≥8 identifying hazardous alcohol use. The AUDIT has been validated in Latin America and in Ecuador (18, 19). Reliability in this sample was good (α = 0.81). Finally, the Health Behavior Inventory (HBI) is a 24-item measure (range 24–120) covering Healthy Eating Habits (EH), Preventive Behaviors (PB), Positive Mental Attitude (PMA), and Health Practices (HP), with higher scores indicating healthier behaviors. The HBI demonstrates strong reliability, with a Cronbach’s α of 0.82 (20). A Spanish adaptation of the HBI, pilot-tested for linguistic clarity and suitability for Ecuadorian medical trainees, was used. The pilot included 15 residents, who provided feedback on item comprehension and survey flow; no substantive modifications were required. Internal consistency for the HBI in this study was excellent (α = 0.91).

### 2.5 Variables and hypotheses

Several sociodemographic and training-related variables were collected to contextualize outcomes. Sociodemographic variables included age (years), gender (male/female), marital status (single/married/divorced/stable union), children (yes/no), religious practice (yes/no), comorbidities (yes/no), living arrangement (alone/with family/with friends), and dependents (yes/no). Training variables included program type (surgical/clinical), specialty (e.g., Cardiology, Orthopedics), year of training, night shifts (yes/no), and weekly work hours. Tobacco use was assessed as a binary variable (“*Do you currently use tobacco*?” including cigarettes or cigars). The selection of these sociodemographic and training variables was informed by evidence from systematic reviews identifying age, gender, workload, specialty, family responsibilities, comorbidities, and lifestyle factors as key determinants of depression, anxiety, and substance use among medical trainees (1, 21, 22).

Based on prior evidence and the variables collected, the study tested three hypotheses:

*H1*: Higher scores on the Health Behavior Inventory (HBI) would be associated with lower odds of depression and anxiety among residents.

*H2*: Residents in surgical programs would have higher odds of hazardous drinking compared with clinical residents.

*H3*: Sociodemographic and training-related factors, including age, gender, dependents, comorbidities, night shifts, and tobacco use, would be associated with differences in depression, anxiety, and hazardous drinking.

### 2.6 Data collection procedures

The questionnaire was distributed via institutional email to all 1,122 residents, inviting them to participate voluntarily. Of these, 307 residents provided informed consent and completed the survey in full, yielding a response rate of 27.4%. Four reminders were sent during the two-month collection period (February 15–March 15, 2025), scheduled to avoid exam stress and reduce bias. Data were collected anonymously, securely stored, and quality-checked; all 307 participants completed the survey without exclusions, and the final dataset was verified for accuracy.

### 2.7 Statistical analysis

Data were analyzed in R (v4.3.2) using *lme4* for mixed-effects modeling (23), *psych* for psychometrics, and *dplyr* for data manipulation (24). We evaluated the distribution of continuous variables (age, weekly work hours, PHQ-9, GAD-7, AUDIT, and HBI scores) using the Kolmogorov–Smirnov test and visual inspection of histograms and Q–Q plots. As these variables showed significant departures from normality (all *p* < 0.05), we summarized them using medians and interquartile ranges (IQR) and used non-parametric tests for group comparisons. Descriptive statistics (medians [IQR] for continuous, frequencies [%] for categorical) summarized characteristics. Mean scores and prevalence (95% CI) for PHQ-9 ≥ 10, GAD-7 ≥ 10, and AUDIT ≥ 8 were calculated. Program types were compared with *t*-tests/chi-square. Spearman correlations assessed associations among PHQ-9, GAD-7, AUDIT, and HBI. Group differences used Wilcoxon (binary) and Kruskal–Wallis (multi-level). Mixed-effects logistic regression modeled PHQ-9 ≥ 10 and GAD-7 ≥ 10 with residency program as a random intercept; a fixed-effects logistic model was used for AUDIT ≥ 8. Predictors included program type, standardized HBI, age, gender, night shifts, comorbidities, tobacco use, and dependents. For each predictor, we report odds ratios (OR) with 95% CIs, Wald χ^2^ statistics, and associated *p*-values, with statistical significance set at *p* < 0.05. Models were fitted with lme4 (‘bobyqa’ optimizer), with Akaike Information Criterion (AIC) and the Bayesian Information Criterion (BIC) guiding selection. The final equations were:

- For PHQ-9 ≥ 10: $\begin{array}{l} \text{logit}(P(Y_{\mathrm{PHQ}}=1))=\beta_{0}+\beta_{1}\cdot \text{Type}\_\text{program}\\ +\beta_{2}\cdot {\mathrm{HBI}}_{\mathrm{std}}+\beta_{3}\cdot {\mathrm{Age}}_{\mathrm{std}}+\beta_{4}\cdot \text{Gender}\\ +\beta_{5}\cdot \text{Dependents}+u_{j} \end{array}$
- For GAD-7 ≥ 10: $\begin{array}{l} \text{logit}(P(Y_{\mathrm{GAD}}=1))=\beta_{0}+\beta_{1}\cdot \text{Type}\_\text{program}\\ +\beta_{2}\cdot {\mathrm{HBI}}_{\mathrm{std}}+\beta_{3}\cdot \text{Gender}+\beta_{5}\cdot \text{Dependents}+u_{j} \end{array}$
- For AUDIT ≥ 8: $\begin{array}{l} \text{logit}(P(Y_{\text{AUDIT}}=1))=\beta_{0}+\beta_{1}\cdot \text{Type}\_\text{program}\\ +\beta_{2}\cdot \mathrm{HBI}\_\mathrm{std}+\beta_{3}\cdot \text{Gender}+\beta_{4}\cdot \text{tobacco}\_\mathrm{use} \end{array}$

Where:

- β_0_: Intercept (baseline log-odds of the outcome)
- β_1_: Effect of program type (1 = Surgical, 0 = Clinical)
- β_2_: Effect of standardized HBI score (M = 0, SD = 1)
- β_3_: Effect of standardized age (M = 0, SD = 1) or Effect of gender (AUDIT only) (1 = Male, 0 = Female)
- β_4_: Effect of gender (1 = Male, 0 = Female) or Effect of tabaco use (AUDIT only) (1 = Yes, 0 = No)
- β_5_: Effect of dependents (1 = Yes, 0 = No)
- *u_j_*: Random intercept for the 18 residency programs (j) (e.g., Cardiology, Orthopedics, General Surgery) (PHQ-9 and GAD-7 only), $u_{j}\sim N(0\sigma_{u}^{2})$ .

Odds ratios (OR) with 95% CIs were reported, with significance at *p* < 0.05.

## 3 Results

The sample consisted of 307 residents with a median age of 33.1 years (IQR 30–36). A total of 169/307 (55.05%) were female. Most participants were single, 169/307 (55.05%), and 284/307 (92.51%) reported practicing a religion. Regarding living arrangements, 143/307 (46.58%) lived alone and 164/307 (53.42%) had financial dependents. Comorbidities were reported by 88/307 (28.66%) participants, of whom 49/307 (15.96%) reported more than one comorbidity. Detailed characteristics are shown in Table 1.

**Table 1:** Characteristics of the sample.

Variable	*n*	%
Age (years)		
Median (IQR)	33.07	(30–36)
Gender		
Female	169	55.05
Male	138	44.94
Marital status		
Single	169	55.05
Married	104	33.88
Divorced	19	6.19
Stable union	15	4.89
Religious practice		
Practice	284	92.51
No practice	23	7.49
Living arrangement		
Alone	143	46.58
Family	70	22.80
Friends	53	17.26
Children		
No	180	58.63
Yes	127	41.37
Number of children		
0	180	58.63
1	69	22.48
2	48	15.64
3	9	2.93
6	1	0.33
Dependents		
No	143	46.58
Yes	164	53.42
Number of dependents		
0	143	46.58
1	70	22.80
2	53	17.26
3	29	9.45
4	10	3.26
5	1	0.33
6	1	0.33
Presence of comorbidities		
Negative	219	71.34
Positive	88	28.66
Cardiovascular	16	18.18
Endocrine and Metabolic	29	32.95
Gastrointestinal	9	10.23
Respiratory and Allergic	7	7.95
Rheumatologic and Musculoskeletal	11	12.50
Neurological and Psychiatric	27	30.68
Ophthalmologic	7	7.95
Others	5	5.68
Total	307	100

The median weekly workload was 70 h (IQR 60–80), and 300/307 (97.72%) residents worked night shifts; 259/307 (84.36%) had two night shifts per week and 32/307 (10.42%) had three. Clinical programs were more common, with 208/307 (67.75%) residents, while 99/307 (32.25%) were in surgical programs. Residents were primarily in their first (122/307; 39.74%) or second (104/307; 33.88%) year of training. Table 2 presents detailed information.

**Table 2:** Training characteristics of the sample.

Variable	*n*	%
Residency program		
Anesthesiology	27	8.79
Cardiology	25	8.14
Colorectal Surgery	9	2.93
Emergency Medicine	29	9.45
Family Medicine	24	7.82
Gastroenterology	31	10.10
General Surgery	9	2.93
Geriatrics	24	7.82
Infectiology	2	0.65
Intensive Care	12	3.91
Internal Medicine	16	5.21
Obstetrics and Gynecology	11	3.58
Orthopedics and Traumatology	12	3.91
Otorhinolaryngology	10	3.26
Palliative Care	17	5.54
Pediatrics	28	9.12
Plastic Surgery	15	4.89
Vascular Surgery	6	1.95
Type of program		
Clinical	208	67.75
Surgical	99	32.25
Program level (year)		
First	122	39.74
Second	104	33.88
Third	77	25.08
Fourth	4	1.30
Work hours per week		
Median (IQR)	70	(60–80)
Night shifts		
No	7	2.28
Yes	300	97.72
Night shifts per week		
0	7	2.28
1	9	2.93
2	259	84.36
3	32	10.42
Total	307	100

### 3.1 Scores

Mean scores for PHQ-9, GAD-7, AUDIT, and HBI were 9.18 (SD = 7.03), 7.86 (SD = 5.76), 3.28 (SD = 3.97), and 69.1 (SD = 13.3), respectively. The mean HBI subcomponent scores were 17.14 (SD = 4.35) for EH, 17.03 (SD = 3.99) for PB, 18.87 (SD = 3.98) for PMA, and 16.03 (SD = 3.48) for HP.

Depression severity, severity of anxiety symptoms, alcohol risk consumption, and health behavior patterns are shown in Table 3. Prevalence rates for clinical cut-offs were 38.76% (95% CI 33.28–44.46) for PHQ-9 ≥ 10, 33.88% (95% CI 28.60–39.47) for GAD-7 ≥ 10, and 12.05% (95% CI 8.63–16.23) for AUDIT ≥ 8. Surgical residents had higher AUDIT scores (*M* = 4.36, SD = 4.76) than clinical residents (Mean = 2.77, SD = 3.42; *p* = 0.003) and greater AUDIT ≥ 8 prevalence (19.19% vs. 8.65%; *p* = 0.014), with no differences in PHQ-9, GAD-7, or HBI (*p* > 0.662).

**Table 3:** Depression severity, severity of anxiety symptoms, alcohol risk consumption, and health behavior patterns.

Variable	*n*	%	95% CI
Depression severity			
Minimal	88	28.66	23.89–33.96
Mild	100	32.57	27.57–38.00
Moderate	57	18.57	14.62–23.30
Moderately severe	29	9.45	6.66–13.24
Severe	33	10.75	7.76–14.71
Anxiety symptoms			
None	97	31.60	26.65–36.99
Mild	106	34.53	29.43–40.01
Moderate	62	20.20	16.09–25.04
Severe	42	13.68	10.28–17.98
Alcohol risk consumption			
Low risk	270	87.95	83.83–91.13
Harmful consumption	29	9.45	6.66–13.24
Dependence	8	2.61	1.33–5.06
Health behavior patterns			
High	13	4.23	2.49–7.11
Average	98	31.92	26.96–37.33
Low	196	63.84	58.33–69.02

### 3.2 Correlations

PHQ-9 and GAD-7 scores were strongly correlated (rho = 0.83, *p* < 0.001), indicating a high degree of co-occurrence between depressive and anxiety symptoms. Healthier behaviors (HBI) were associated with lower PHQ-9 and GAD-7 scores (rho = −0.46 and −0.36, respectively, *p* < 0.001). A weak positive association was observed between PHQ-9 and AUDIT scores (rho = 0.12, *p* = 0.036), while AUDIT and HBI were uncorrelated (rho = −0.04, *p* = 0.52).

The HBI subscales exhibited significant negative correlations with PHQ-9 and GAD-7 scores, with PMA showing the strongest associations (PHQ-9: rho = −0.49, *p* < 0.001; GAD-7: rho = −0.42, *p* < 0.001), followed by HP (PHQ-9: rho = −0.42, *p* < 0.001; GAD-7: rho = −0.36, *p* < 0.001), EH (PHQ-9: rho = −0.38, *p* < 0.001; GAD-7: rho = −0.30, *p* < 0.001), and PB (PHQ-9: rho = −0.26, *p* < 0.001; GAD-7: rho = −0.15, *p* = 0.009). In contrast, no significant associations were found between HBI subscales and AUDIT scores (rho range: −0.06 to 0.002, *p* > 0.26), suggesting that health-promoting behaviors were unrelated to alcohol consumption patterns.

Biserial correlations with clinical cut-offs (PHQ-9 ≥ 10, GAD-7 ≥ 10, AUDIT ≥ 8) mirrored these patterns, with positive mental attitudes being the strongest protective factor against moderate-to-severe depression (*r* = −0.41, *p* < 0.001) and anxiety (*r* = −0.31, *p* < 0.001). In contrast, AUDIT ≥ 8 showed no significant associations (r range: −0.10 to −0.05, *p* > 0.08).

**Figure 1:**
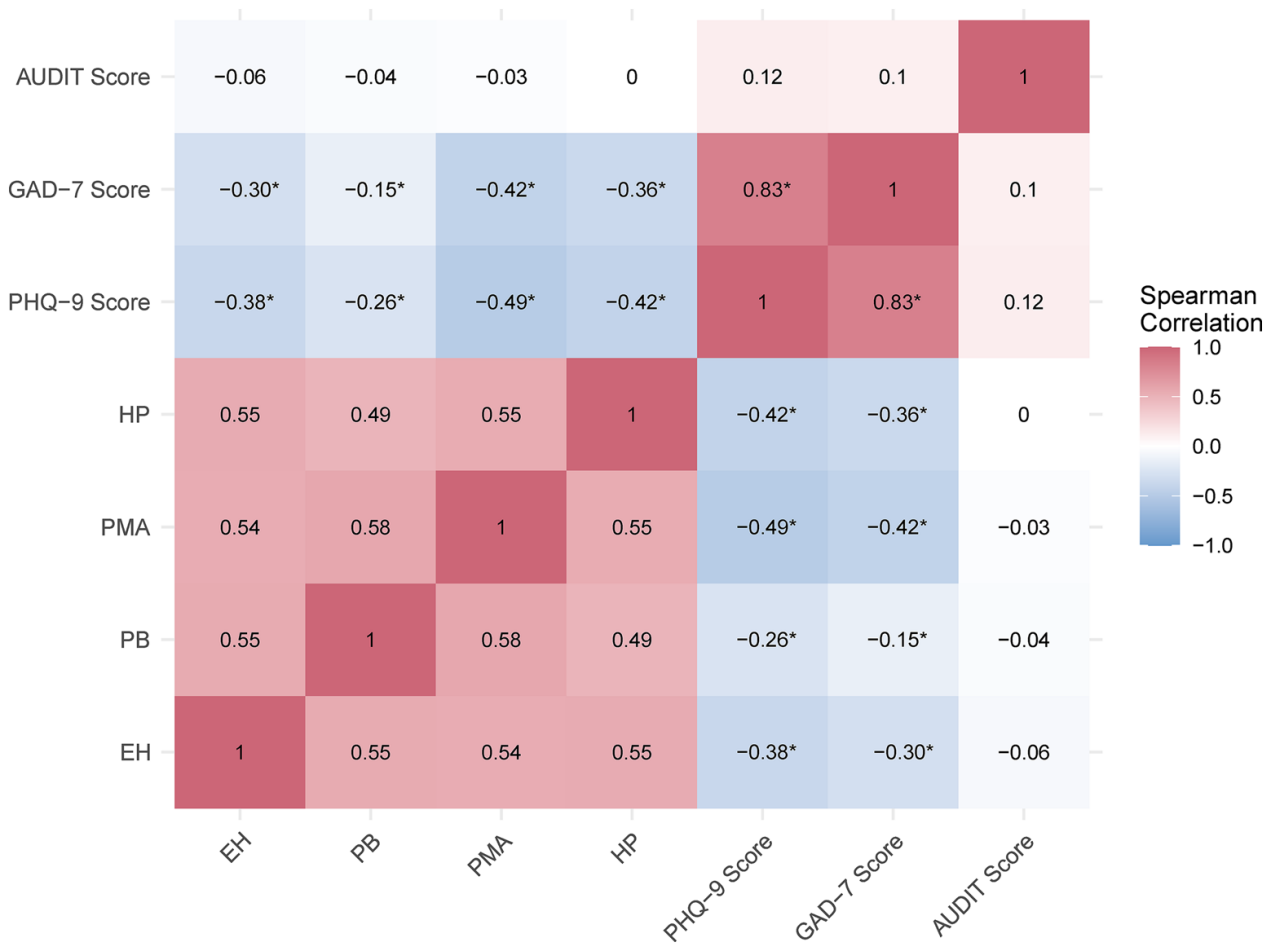
Spearman correlation heatmap of HBI subscales and mental health measures. Heatmap of Spearman correlations between HBI subscales—EH (Healthy Eating), PB (Preventive Behavior), PMA (Positive Mental Attitude), HP (Health Practices)—and mental health scores (PHQ-9, GAD-7, AUDIT). Color intensity reflects correlation strength (blue = negative, purple = positive); values show rho. All HBI subscales correlations with PHQ-9 and GAD-7 were significant (*).

These relationships are visually summarized in Figure 1, where the strong positive correlation between PHQ-9 and GAD-7 (rho = 0.83) is represented by intense reddish-purple shading. Conversely, the negative correlations between HBI subscales and both PHQ-9 and GAD-7 appear in varying shades of blue, with deeper blue tones highlighting the strongest associations—such as PMA (rho = −0.49 for PHQ-9, rho = −0.42 for GAD-7). In contrast, the lack of association between AUDIT and HBI subscales is reflected in off-white cells, indicating correlations close to zero.

**Figure 2:**
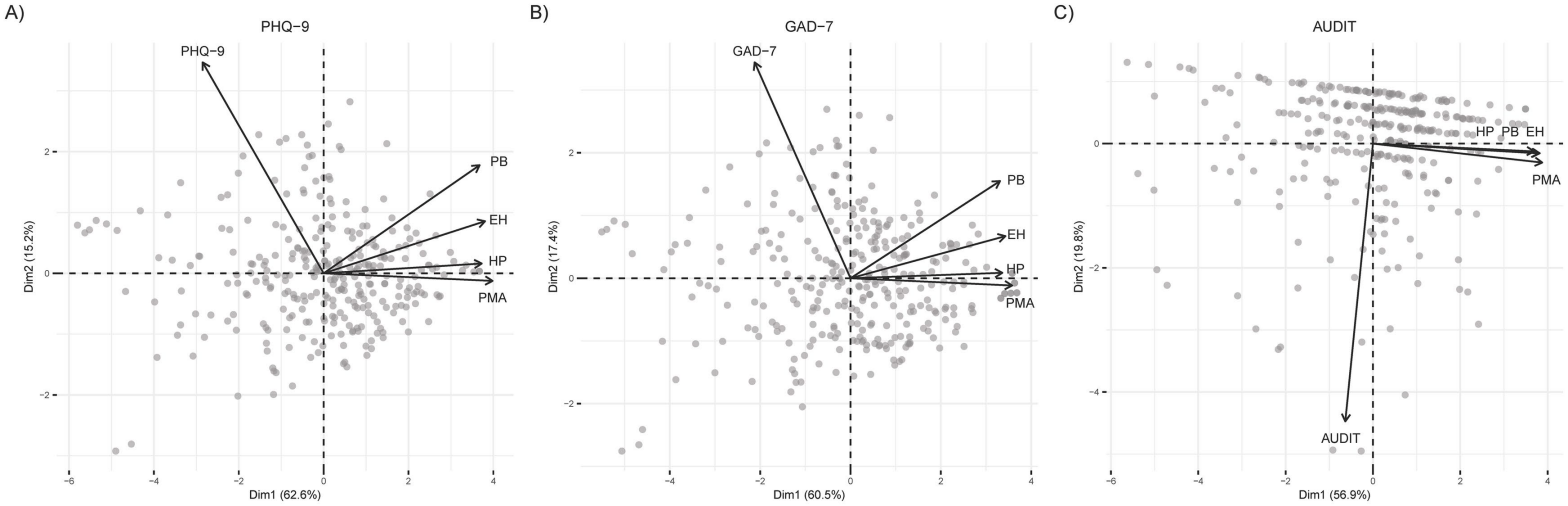
Principal component analysis biplots of health behaviors and mental health measures. Biplots display variable loadings of HBI subscales—EH (Healthy Eating), PB (Preventive Behavior), PMA (Positive Mental Attitude), and HP (Health Practices)—alongside **(A)** PHQ-9, **(B)** GAD-7, and **(C)** AUDIT. PC1 and PC2 together explain 79.8, 79.7, and 84.8% of variance in panels A, B, and C, respectively. Arrows indicate variable contributions to each principal component.

Figure 2 further illustrates these patterns using PCA biplots, which show the relationships between HBI subscales and (A) PHQ-9, (B) GAD-7, and (C) AUDIT. In Figures 2A,B, the HBI vectors (EH, PB, PMA, HP) project in the opposite direction of PHQ-9 and GAD-7, visually reinforcing their negative associations. PMA shows the strongest contribution, consistent with its higher correlations. In contrast, the near-orthogonal orientation of AUDIT in Figure 2C highlights its lack of association with the HBI subscales.

### 3.3 Group comparisons

Wilcoxon tests compared binary groups; Kruskal-Wallis tested multi-level variables. Males had higher AUDIT scores (*p* < 0.001). Age tertiles (youngest, *n* = 103; middle, *n* = 102; oldest, *n* = 102) showed trends for higher GAD-7 (*p* = 0.056) and AUDIT (*p* = 0.079) in the youngest tertile. Children (Yes, *n* = 127) increased HBI (*p* = 0.012); dependents (Yes, *n* = 164) lowered PHQ-9 (*p* = 0.007) and raised HBI (*p* = 0.001). Comorbidities (Yes, *n* = 88) increased PHQ-9 (*p* = 0.010) and GAD-7 (*p* = 0.046). Tobacco use (Yes, *n* = 37) raised AUDIT (*p* = 0.001) and lowered HBI (*p* = 0.009). Night shifts (Yes, *n* = 300) increased GAD-7 (*p* = 0.015) and reduced HBI (*p* = 0.020). Religion and marital status showed no differences (*p* > 0.077).

### 3.4 Mixed-effects logistic regression

Mixed-effects models (random effect: residency program) and a fixed-effects model (AUDIT) were used to examine predictors of PHQ-9 ≥ 10, GAD-7 ≥ 10, and AUDIT ≥ 8. Table 4 summarizes these findings, with additional details on the ORs and 95% CIs.

**Table 4:** Predictors for depression, anxiety, and alcohol consumption risk.

Score	Predictor	OR (95% CI)	χ^2^	*p* value
PHQ-9 ≥ 10				
	Type of program (surgical)	0.91 (0.48–1.71)	0.09	0.074
	HBI score (standardized)	0.34 (0.24–0.47)	39.53	**<0.001**
	Gender (male)	0.54 (0.31–0.95)	4.61	**0.032**
	Age (standardized)	0.71 (0.53–0.96)	5.11	**0.024**
	Dependents	1.64 (0.93–2.90)	2.89	0.089
GAD-7 ≥ 10				
	Type of program (surgical)	0.87 (0.46–1.66)	0.17	0.676
	HBI score (standardized)	0.52 (0.39–0.69)	20.47	**<0.001**
	Gender (male)	0.67 (0.39–1.16)	2.08	0.150
	Age (standardized)	0.65 (0.49–0.87)	8.13	**0.004**
	Dependents	1.54 (0.88–2.71)	2.27	0.132
AUDIT ≥ 8				
	Type of program (surgical)	2.21 (1.08–4.54)	4.74	**0.029**
	HBI score (standardized)	0.83 (0.59–1.18)	1.17	0.280
	Gender (male)	1.23 (0.57–2.64)	0.28	0.599
	Tobacco use (yes)	2.64 (1.02–6.56)	4.22	**0.040**

Values in bold means they are statistically significant.

For PHQ-9 ≥ 10 (SD = 0.28), significant predictors included HBI (OR = 0.34, 95% CI 0.24–0.47, *p* < 0.001), male gender (OR = 0.54, 95% CI 0.31–0.95, *p* = 0.032), and older age (OR = 0.71, 95% CI 0.53–0.96, *p* = 0.024), which were associated with lower odds of depression. For GAD-7 ≥ 10 (SD = 0.32), HBI (OR = 0.52, 95% CI 0.39–0.69, *p* < 0.001) and age (OR = 0.65, 95% CI 0.49–0.87, *p* = 0.004) were associated with lower odds of anxiety, while gender and tobacco use showed marginal effects (*p* > 0.092).

For AUDIT ≥ 8, surgical program (OR = 2.21, 95% CI 1.08–4.54, *p* = 0.029) and tobacco use (OR = 2.64, 95% CI 1.02–6.56, *p* = 0.040) were associated with higher odds of alcohol consumption risk.

## 4 Discussion

This study provides valuable insights into the mental health challenges faced by residents in Ecuador, offering comparisons with similar research across Latin America. Our findings converge with some regional estimates while diverging in others, underscoring the complex interplay of individual, clinical, and institutional determinants of mental health during residency. To contextualize our results, we contrast them with evidence from neighboring countries.

In Argentina, 48% of cardiology residents reported depression, linked to long hours and financial strain, with 60% holding second jobs due to low income (25). While our prevalence and workload were similar, financial stress did not emerge as a significant factor in our cohort. This is notable because, unlike Argentina, most Ecuadorian residents do not receive stipends or salaries from hospitals or the government and must instead pay their training fees out-of-pocket. Despite this considerable financial burden, economic strain was not associated with depression in our sample, suggesting that other factors, such as workload intensity, limited mental health support, or lifestyle behaviors, may play a more prominent role in shaping residents’ mental well-being.

Although residency in Mexico is also characterized by long work hours and high service demands the study by Pérez Cruz (26) reported lower prevalence of depression (16.3% mild to moderate). This difference may reflect the institutional context of their single-site cohort, where emotional exhaustion, reduced accomplishment, and comorbidities were the primary predictors. These determinants differ from those identified in our broader, multi-specialty sample in Ecuador, suggesting that institutional variation and program-specific factors may shape mental health outcomes differently across settings.

Another Mexican study reported low rates of moderate-to-severe anxiety (11.7%) and depression (13%), with increased prevalence in gynecology-obstetrics and emergency medicine (27). These proportions contrast with our higher rates, possibly reflecting differences in institutional support, local resilience-building strategies, or sample composition. Still, the strong association between anxiety and depression observed in that study (*r* = 0.711, *p* < 0.001) mirrored our findings (rho = 0.83, *p* < 0.001), highlighting their well-documented co-occurrence under training pressures.

In Brazil, depression and anxiety rates of 21 and 41% were linked to burnout and low social skills (28), aligning with our anxiety prevalence but differing in depression. Moreover, in Brazil during the COVID-19 pandemic, rates increased dramatically to 65.8% for depression, 49.7% for anxiety, and 49.2% for burnout, driven by high patient mortality, overwhelming service demands, and perceived unpreparedness (29). Similar evidence links preparedness and stressful work to poorer mental health (30, 31). Our post-pandemic prevalence was lower yet remained within the upper global range. A meta-analysis reported 28.8% depression among residents, rising to 43.2% in recent studies (21), situating our findings within international trends. In the United States, burnout affects 60% and depressive symptoms 51% of residents, largely due to excessive workloads and poor work-life balance (22). Although Ecuadorian residents reported similarly intense schedules (median 70 h per week with frequent night shifts), their depression prevalence was slightly lower, suggesting that cultural norms, coping strategies, or institutional context may modulate the impact of long working hours.

Lifestyle and preventive health behaviors among Latin American residents remain insufficiently characterized. In Colombia, only 14.4% of physicians engaged in adequate physical activity, 13.6% reported poor self-care, and 9% inadequate sleep (32). Our residents showed even lower overall HBI scores, indicating pervasive unhealthy behaviors that may be exacerbated by the demands of residency. In Peru, despite 57.5% reporting stress, 85.5% engaged in healthy practices, suggesting that lifestyle behaviors may buffer psychological distress (33). In contrast, our findings show that poor health behaviors were common and strongly associated with depression and anxiety, reinforcing their protective role in mental health. In the United States, although preventive screenings were common, many physicians reported minimal physical activity (35%), skipped breakfast (27%), or screened positive for alcohol misuse (6%) (34). This gap between medical knowledge and personal practices was also present in our sample, where despite their training, most residents did not achieve high healthy-behavior scores.

Evidence from Israel similarly highlights this pattern: 71% of physicians did not meet physical activity recommendations, 79% had poor nutrition, and 57% lacked a personal physician (35). Poor nutrition, inadequate sleep, and perceived poor health were associated with greater stress (*p* < 0.001). Our residents showed a similar profile, with poor health behaviors strongly linked to depression and anxiety. These parallels suggest that the failure to translate medical knowledge into personal health practices is a consistent global phenomenon. By documenting this association among Ecuadorian residents, our study contributes evidence from a Latin American context in which such data have been limited and underscores the global relevance of lifestyle promotion during residency.

Mental health symptoms often emerge early in residency. In the United States., residents reported poorer habits than students or attendings, with 34% sleeping ≤6 h and 53% experiencing severe stress (36). Another study showed 29% with depressive symptoms, 6% with harmful alcohol use, and 5% using sedatives without prescription (37). In Spain, 80% of first-year residents adopted at least one unhealthy habit, mainly low physical activity (42.1%) and sleep deprivation (42.4%) (38). Our results mirror these findings, demonstrating that younger age and unhealthy behaviors were independent predictors of depression and anxiety. This reinforces the notion that early residency represents a vulnerable period, where new responsibilities and demanding schedules may precipitate the onset of mental health symptoms and maladaptive behaviors. Early preventive strategies are therefore essential.

Hazardous drinking among healthcare professionals is a growing concern, with alcohol often used as a coping strategy (39). In Brazil, 32% of physicians reported alcohol use, with higher rates in men (40, 41). In Mexico, harmful consumption reached 55.6% among early-year residents, strongly correlated with anxiety (*r* = 0.862, *p* < 0.001) (42), while another study found higher use in final-year residents, likely due to stress (43). In contrast, our findings showed no gender differences and no correlation with anxiety; however, hazardous drinking was associated with depression. This divergence may reflect cultural norms, reporting differences, or sample characteristics, as well as the comparatively lower anxiety prevalence in our cohort. Collectively, these findings suggest that hazardous drinking does not follow a uniform pattern across settings and that its relationship with mental health may vary by context, highlighting the importance of locally tailored prevention strategies (44–46).

The high prevalence of depression, anxiety, and unhealthy behaviors among Ecuadorian residents likely reflects the intensity of training, including high patient loads, extended work hours, and frequent 24-h shifts. Although regulations mandate 64 clinical and 16 academic hours weekly, residents often exceed 100 (47, 48). Yet, workload and night shifts were not significantly linked to mental health in our study. This contrasts with literature consistently linking long duty hours with burnout, depression, and medical errors (49). One explanation may be that the impact of workload arises less from the sheer number of hours than from the nature of responsibilities during those hours, such as decision-making pressure, lack of autonomy, and emotional strain. Another possibility is that demanding schedules are normalized within the local context, making their statistical effects less apparent when compared with stronger predictors, such as unhealthy lifestyle behaviors. Future research should therefore evaluate both structural and cultural dimensions of residency training rather than focusing solely on quantitative workload metrics.

The mental health challenges observed in our study underscore the need for integrated support systems, including routine monitoring, access to mental health professionals, stress management interventions, and peer support programs. Complementary measures should address the structural drivers of distress, such as excessive workloads and insufficient legal protections, particularly in high-stress specialties (50). Beyond individual well-being, strengthening residents’ mental health is a public health priority, as elevated rates of depression, anxiety, and hazardous drinking may compromise patient safety and threaten the sustainability of healthcare systems. These recommendations align with WHO calls to promote workforce well-being as a foundation for resilient health systems (6, 51).

This study has several strengths. The use of validated instruments (PHQ-9, GAD-7, AUDIT, HBI) allowed standardized measurement and comparability with regional and international data. Random sampling and high participation enhanced representativeness, while assessing multiple domains, mental health, alcohol use, and health behaviors, provided a comprehensive perspective consistent with global calls for holistic evaluations in residency training.

Several limitations should be acknowledged. The single-institution design may limit generalizability, although residents trained in multiple cities partly mitigates this concern (52). Reliance on self-reports raises the risk of underreporting due to stigma or social desirability, particularly for alcohol use and mental health symptoms. Selection bias is also possible if more distressed residents chose not to participate, potentially leading to underestimation of prevalence. We did not assess other relevant determinants of mental health, such as personality traits, sleep quality, or the use of stimulants or sedative medications (e.g., benzodiazepines), which may further influence resident well-being and should be explored in future studies. Finally, the cross-sectional design precludes causal inference, and the lack of longitudinal follow-up limits understanding of symptom trajectories over time (21). Future research in Ecuador should also examine specialty-specific differences, organizational culture, and the impact of extreme duty hours and shift structures, particularly in settings where residents may work more than 100 h per week, on mental health and health-promoting behaviors.

## Data Availability

The datasets used and/or analyzed during the current study are available through the relevant author upon reasonable request.
